# Aqua­(hippurato)bis­(1,10-phenanthroline)cobalt(II) nitrate monohydrate

**DOI:** 10.1107/S1600536810040602

**Published:** 2010-10-20

**Authors:** Gui-Quan Guo, Ji-Hua Deng, Jing Chen

**Affiliations:** a College of Chemistry & Chemical Engineering, Anyang Normal University, Anyang, Henan 455000, People’s Republic of China; b Key Laboratory of Jiangxi University for Applied Chemistry and Chemical Biology, College of Chemistry & Bioengineering, Yichun University, Yichun, Jiangxi 336000, People’s Republic of China

## Abstract

In the title compound, [Co(C_9_H_8_NO_3_)(C_12_H_8_N_2_)_2_(H_2_O)]NO_3_·H_2_O, the Co^II^ atom is six-coordinated by a carboxylate O atom of the hippurate (Hc) anion, a water O atom and four N atoms from two 1,10-phenanthroline ligands in a distorted octa­hedral geometry. The uncoordinated O atom of the hippuric acid anion is involved in an intra­molecular hydrogen bond to the coordinated water mol­ecule. The crystal packing is stabilized by inter­molecular O—H⋯O hydrogen bonds involving the Hc anions, the coordinated water mol­ecule, the nitrate anion and the uncoordinated water mol­ecule.

## Related literature

For complexes based on hippuric acid, see: Antolini *et al.* (1982 ▶); Brown & Trefonas (1973 ▶); Grewe *et al.* (1982 ▶); Guo, Chen *et al.* (2006 ▶); Guo, Wang *et al.* (2006 ▶); Morelock *et al.* (1979 ▶, 1982 ▶).
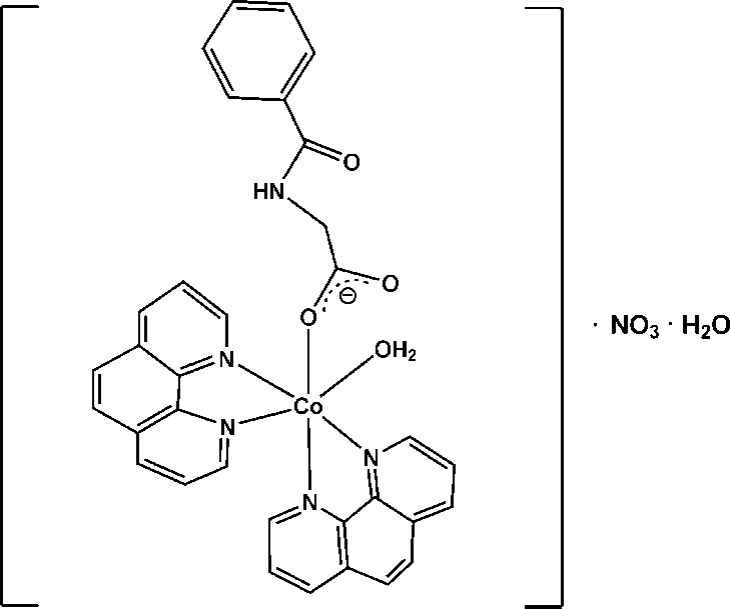


## Experimental

### 

#### Crystal data

[Co(C_9_H_8_NO_3_)(C_12_H_8_N_2_)_2_(H_2_O)]NO_3_·H_2_O
*M* *_r_* = 695.54
Monoclinic, 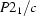

*a* = 9.935 (2) Å
*b* = 13.991 (3) Å
*c* = 23.162 (5) Å
β = 91.37 (3)°
*V* = 3218.7 (11) Å^3^
*Z* = 4
Mo *K*α radiation
μ = 0.60 mm^−1^
*T* = 291 K
0.20 × 0.18 × 0.17 mm

#### Data collection

Bruker P4 diffractometer
Absorption correction: multi-scan (*SADABS*; Sheldrick, 1996 ▶) *T*_min_ = 0.890, *T*_max_ = 0.906
9187 measured reflections
5493 independent reflections
3848 reflections with *I* > 2σ(*I*)
*R*_int_ = 0.043

#### Refinement

*R*[*F* ^2^ > 2σ(*F* ^2^)] = 0.066
*wR*(*F* ^2^) = 0.143
*S* = 1.09
5493 reflections
445 parameters
3 restraints
H atoms treated by a mixture of independent and constrained refinement
Δρ_max_ = 0.53 e Å^−3^
Δρ_min_ = −0.30 e Å^−3^

### 

Data collection: *SMART* (Bruker, 1998 ▶); cell refinement: *SAINT* (Bruker, 1998 ▶); data reduction: *SAINT*; program(s) used to solve structure: *SHELXS97* (Sheldrick, 2008 ▶); program(s) used to refine structure: *SHELXL97* (Sheldrick, 2008 ▶); molecular graphics: *SHELXTL* (Sheldrick, 2008 ▶); software used to prepare material for publication: *SHELXTL*.

## Supplementary Material

Crystal structure: contains datablocks I, global. DOI: 10.1107/S1600536810040602/kp2275sup1.cif

Structure factors: contains datablocks I. DOI: 10.1107/S1600536810040602/kp2275Isup2.hkl

Additional supplementary materials:  crystallographic information; 3D view; checkCIF report

## Figures and Tables

**Table 1:** Selected bond lengths (Å)

Co1—O2	2.076 (3)
Co1—O4	2.106 (4)
Co1—N5	2.130 (3)
Co1—N2	2.133 (3)
Co1—N3	2.142 (3)
Co1—N4	2.173 (3)

**Table 2:** Hydrogen-bond geometry (Å, °)

*D*—H⋯*A*	*D*—H	H⋯*A*	*D*⋯*A*	*D*—H⋯*A*
O8—H8*E*⋯O5	0.87	2.46	3.084 (7)	129
O8—H8*E*⋯O7	0.87	2.24	2.982 (7)	143
O4—H4*F*⋯O2	0.85 (7)	2.48 (7)	2.876 (5)	110 (5)
O4—H4*F*⋯O3	0.85 (7)	1.87 (7)	2.696 (5)	162 (7)

**Fig. 1.**
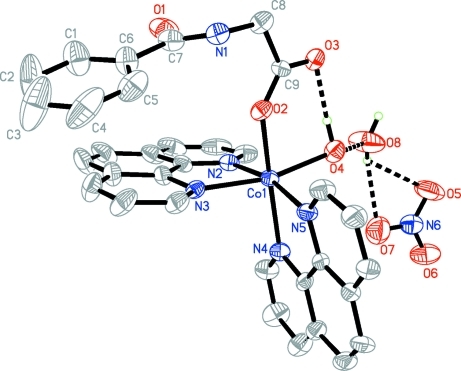
The molecular structure of (I) (hydrogen bonds are shown by dashed lines).

**Fig. 2.**
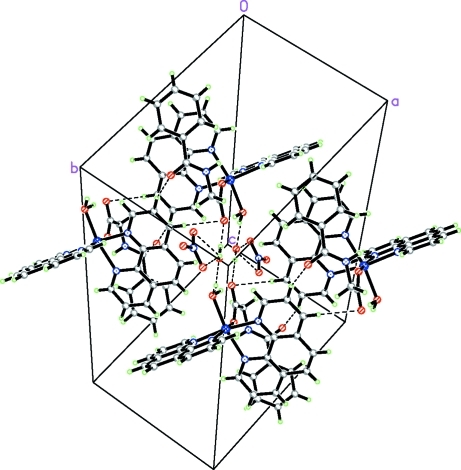
The crystal packing of (I) stabilised by intermolecular hydrogen bonds (dashed lines).

## supplementary crystallographic information

### Comment

Hippuric acid (Hc) is a naturally occurring carboxylic acid found in the urine of most mammals. Several decades ago, hippuric acid was testified as a good building units for metal complexes with desirable magnetic properties, due to the carboxylic group of hippuric acid can act as donor to link metal centers. To date, the synthesis, crystal structures, and related property studying of Ni(II), Fe(II), Zn(II), Cu(II) complexes based on hippuric acid have been reported (Antolini, *et al.*, 1982; Brown, *et al.*, 1973; Grewe, *et al.*, 1982; Guo, Wang, Chen, *et al.*, 2006; Guo, Chen, Wang, *et al.*, 2006; Morelock, Good, Trefonas, Karraker *et al.*, 1979; Morelock, Good, Trefonas, Majeste *et al.*, 1982). Herein, we present the synthesis and crystal structure of its Co(II) complex.

The title compound, (I), is  a mononuclear complex, consisting of one [Co(Hc)(phen)_2_(H_2_O)]^+^ unit, one NO_3_^-^ anion, and one crystal H_2_O molecule (Fig. 1). The Co(II) ion is six-coordinated by  O atoms from one Hc anion and one water molecule, four N atoms of two 1,10-phen ligands in  a shape of distorted octahedron. The atoms N2, N3, N5, and O4 are located in the equatorial plane whereas O2 and N4 occupy the axial positions. The bond distances and angles around Co atom reveal a distorted octahedron (bite-chelating angles are 77.2 and 77.822 °). The Hc anion acts as a monodentate ligand, with one of the carboylic oxygen atoms coordinated to the Co(II) ions, while the amide O and imine N atoms remain uncoordinated.

The coordinated water molecule and the crystal water molecule donate H atom to the nitrate anion and to the Hc anion ligand to form O—H···O hydrogen bonds. In addition, the imine group of the Hc anion also donate H atom to the nitrate to form N—H···O hydrogen bonds (Table 1, Fig. 2).

### Experimental

Hippuric acid (1.0 mmol) , Co(NO_3_)_2_.6H_2_ (O1.0 mmol)  and 1,10-phenanthroline (2.0 mmol) were dissolved in a water-ethanol mixture(*v*/*v*=1:4; 25 ml). The obtained solution was continously stirred, and its pH was adjusted to 6–7 by 1.0 mol *L*^-1^ NaOH aqueous solution. Then the mixture was further stirred for *ca* 2 h at room temperature and filtered. The resultant filtrate was left to stand for slow evaporation at room temperature. Dark-red single crystals of (I) suitable for X-ray diffraction analysis were obtained after two weeks (yield 66%).

### Refinement

Hydrogen atoms attached to carbon atoms and nitrogen atoms were positioned geometrically and treated as riding, with C—H = 0.93 Å, N—H = 0.86 Å, and *U*_iso_(H) = 1.2*U*_eq_(C or N). The hydrogen atoms of the coordination water molecules and lattice water molecules were assigned in the difference Fourier maps and refined isotropically.

### Crystal data

**Table:**

[Co(C_9_H_8_NO_3_)(C_12_H_8_N_2_)_2_(H_2_O)]NO_3_·H_2_O	*F*(000) = 1436
*M**_r_* = 695.54	*D*_x_ = 1.435 Mg m^−^^3^
Monoclinic, *P*2_1_/*c*	Mo *K*α radiation, λ = 0.71073 Å
Hall symbol: -P 2ybc	Cell parameters from 1226 reflections
*a* = 9.935 (2) Å	θ = 1.7–25.5°
*b* = 13.991 (3) Å	µ = 0.60 mm^−^^1^
*c* = 23.162 (5) Å	*T* = 291 K
β = 91.37 (3)°	Prismatic, dark-red
*V* = 3218.7 (11) Å^3^	0.20 × 0.18 × 0.17 mm
*Z* = 4	

### Data collection

**Table:**

Bruker P4 diffractometer	5493 independent reflections
Radiation source: fine-focus sealed tube	3848 reflections with *I* > 2σ(*I*)
graphite	*R*_int_ = 0.043
ω scans	θ_max_ = 25.5°, θ_min_ = 1.7°
Absorption correction: multi-scan (*SADABS*; Sheldrick, 1996)	*h* = 0→12
*T*_min_ = 0.890, *T*_max_ = 0.906	*k* = −16→16
9187 measured reflections	*l* = −28→28

### Refinement

**Table:**

Refinement on *F*^2^	Secondary atom site location: difference Fourier map
Least-squares matrix: full	Hydrogen site location: inferred from neighbouring sites
*R*[*F*^2^ > 2σ(*F*^2^)] = 0.066	H atoms treated by a mixture of independent and constrained refinement
*wR*(*F*^2^) = 0.143	*w* = 1/[σ^2^(*F*_o_^2^) + (0.0588*P*)^2^ + 1.9834*P*] where *P* = (*F*_o_^2^ + 2*F*_c_^2^)/3
*S* = 1.09	(Δ/σ)_max_ = 0.001
5493 reflections	Δρ_max_ = 0.53 e Å^−^^3^
445 parameters	Δρ_min_ = −0.30 e Å^−^^3^
3 restraints	Extinction correction: *SHELXL97* (Sheldrick, 2008), Fc^*^=kFc[1+0.001xFc^2^λ^3^/sin(2θ)]^-1/4^
Primary atom site location: structure-invariant direct methods	Extinction coefficient: 0.0026 (5)

### Special details

**Table:**

Geometry. All e.s.d.'s (except the e.s.d. in the dihedral angle between two l.s. planes) are estimated using the full covariance matrix. The cell e.s.d.'s are taken into account individually in the estimation of e.s.d.'s in distances, angles and torsion angles; correlations between e.s.d.'s in cell parameters are only used when they are defined by crystal symmetry. An approximate (isotropic) treatment of cell e.s.d.'s is used for estimating e.s.d.'s involving l.s. planes.
Refinement. Refinement of *F*^2^ against ALL reflections. The weighted *R*-factor *wR* and goodness of fit *S* are based on *F*^2^, conventional *R*-factors *R* are based on *F*, with *F* set to zero for negative *F*^2^. The threshold expression of *F*^2^ > σ(*F*^2^) is used only for calculating *R*-factors(gt) *etc*. and is not relevant to the choice of reflections for refinement. *R*-factors based on *F*^2^ are statistically about twice as large as those based on *F*, and *R*- factors based on ALL data will be even larger.

### Fractional atomic coordinates and isotropic or equivalent isotropic displacement parameters (Å^2^)

**Table:**

	*x*	*y*	*z*	*U*_iso_*/*U*_eq_	
Co1	0.22952 (5)	0.25150 (4)	0.93865 (2)	0.03887 (18)	
N1	0.2492 (4)	0.0562 (3)	0.77418 (15)	0.0519 (10)	
N2	0.0799 (4)	0.1728 (2)	0.98300 (14)	0.0457 (9)	
N3	0.0537 (3)	0.2743 (2)	0.88506 (14)	0.0427 (8)	
N4	0.1985 (4)	0.3762 (2)	0.99264 (15)	0.0474 (9)	
N5	0.3523 (3)	0.3570 (2)	0.89962 (15)	0.0461 (9)	
N6	0.4811 (5)	0.2629 (3)	0.2140 (2)	0.0739 (12)	
O1	0.0673 (4)	−0.0126 (3)	0.81183 (15)	0.0887 (12)	
O2	0.2726 (3)	0.15136 (19)	0.87560 (12)	0.0500 (7)	
O3	0.4124 (3)	0.0438 (2)	0.91520 (12)	0.0532 (8)	
O4	0.3952 (4)	0.1959 (3)	0.98609 (17)	0.0594 (9)	
O5	0.5553 (6)	0.1969 (3)	0.2076 (2)	0.135 (2)	
O6	0.4928 (5)	0.3168 (3)	0.25517 (19)	0.1063 (15)	
O7	0.3906 (5)	0.2794 (3)	0.1776 (2)	0.1195 (16)	
C1	−0.1062 (7)	0.0710 (5)	0.7295 (3)	0.098 (2)	
H1A	−0.1383	0.0200	0.7510	0.118*	
C2	−0.1936 (10)	0.1199 (8)	0.6925 (4)	0.143 (4)	
H2A	−0.2833	0.1015	0.6890	0.171*	
C3	−0.1478 (13)	0.1941 (9)	0.6619 (4)	0.162 (6)	
H3A	−0.2068	0.2271	0.6374	0.194*	
C4	−0.0191 (11)	0.2214 (6)	0.6660 (3)	0.128 (3)	
H4A	0.0100	0.2729	0.6442	0.153*	
C5	0.0727 (7)	0.1734 (5)	0.7027 (2)	0.0872 (18)	
H5A	0.1622	0.1927	0.7053	0.105*	
C6	0.0286 (5)	0.0969 (4)	0.7350 (2)	0.0635 (13)	
C7	0.1167 (6)	0.0431 (3)	0.77715 (19)	0.0585 (13)	
C8	0.3383 (5)	0.0180 (3)	0.81921 (18)	0.0573 (12)	
H8A	0.4289	0.0149	0.8046	0.069*	
H8B	0.3103	−0.0467	0.8280	0.069*	
C9	0.3408 (4)	0.0765 (3)	0.87478 (17)	0.0416 (10)	
C10	0.0916 (6)	0.1286 (3)	1.0340 (2)	0.0655 (14)	
H10A	0.1744	0.1286	1.0536	0.079*	
C11	−0.0189 (8)	0.0818 (4)	1.0587 (2)	0.0839 (19)	
H11A	−0.0094	0.0534	1.0949	0.101*	
C12	−0.1382 (7)	0.0784 (4)	1.0298 (3)	0.0837 (19)	
H12A	−0.2112	0.0473	1.0458	0.100*	
C13	−0.1515 (5)	0.1217 (3)	0.9762 (3)	0.0679 (15)	
C14	−0.0405 (4)	0.1707 (3)	0.9552 (2)	0.0483 (11)	
C20	−0.2734 (6)	0.1177 (4)	0.9418 (4)	0.095 (2)	
H20A	−0.3472	0.0846	0.9555	0.114*	
C15	−0.0536 (4)	0.2210 (3)	0.90145 (19)	0.0462 (11)	
C16	−0.1735 (5)	0.2160 (3)	0.8682 (3)	0.0664 (14)	
C17	−0.1808 (7)	0.2669 (4)	0.8170 (2)	0.0786 (18)	
H17A	−0.2583	0.2642	0.7937	0.094*	
C18	−0.0740 (7)	0.3207 (4)	0.8007 (2)	0.0808 (18)	
H18A	−0.0785	0.3562	0.7667	0.097*	
C19	0.0429 (5)	0.3219 (3)	0.83589 (19)	0.0609 (13)	
H19A	0.1161	0.3579	0.8241	0.073*	
C21	−0.2826 (6)	0.1609 (4)	0.8902 (4)	0.090 (2)	
H21A	−0.3616	0.1551	0.8682	0.108*	
C22	0.1209 (5)	0.3858 (3)	1.0377 (2)	0.0638 (13)	
H22A	0.0696	0.3337	1.0488	0.077*	
C23	0.1115 (6)	0.4693 (4)	1.0694 (2)	0.0798 (17)	
H23A	0.0550	0.4728	1.1007	0.096*	
C24	0.1866 (6)	0.5465 (4)	1.0538 (2)	0.0757 (16)	
H24A	0.1821	0.6028	1.0750	0.091*	
C25	0.2702 (5)	0.5411 (3)	1.0064 (2)	0.0570 (12)	
C26	0.2721 (4)	0.4529 (3)	0.97703 (18)	0.0449 (10)	
C27	0.3558 (4)	0.4429 (3)	0.92735 (18)	0.0431 (10)	
C28	0.4375 (5)	0.5187 (3)	0.9108 (2)	0.0538 (12)	
C29	0.5179 (5)	0.5031 (4)	0.8623 (2)	0.0714 (15)	
H29A	0.5738	0.5515	0.8494	0.086*	
C30	0.5141 (5)	0.4184 (4)	0.8347 (2)	0.0706 (15)	
H30A	0.5677	0.4079	0.8029	0.085*	
C31	0.4292 (5)	0.3463 (3)	0.8541 (2)	0.0601 (13)	
H31A	0.4265	0.2885	0.8343	0.072*	
C32	0.3542 (6)	0.6170 (3)	0.9870 (3)	0.0743 (16)	
H32A	0.3532	0.6752	1.0064	0.089*	
C33	0.4342 (6)	0.6065 (3)	0.9417 (3)	0.0735 (16)	
H33A	0.4882	0.6572	0.9304	0.088*	
O8	0.4361 (6)	0.1252 (3)	0.09134 (17)	0.133 (2)	
H8F	0.4828	0.0745	0.0893	0.200*	
H8E	0.4121	0.1459	0.1249	0.200*	
H1G	0.289 (8)	0.100 (5)	0.754 (3)	0.160*	
H4F	0.394 (7)	0.141 (5)	0.970 (3)	0.14 (3)*	
H4E	0.395 (6)	0.178 (4)	1.022 (3)	0.09 (2)*	

### Atomic displacement parameters (Å^2^)

**Table:**

	*U*^11^	*U*^22^	*U*^33^	*U*^12^	*U*^13^	*U*^23^
Co1	0.0449 (3)	0.0334 (3)	0.0384 (3)	−0.0027 (3)	0.0039 (2)	−0.0031 (3)
N1	0.069 (3)	0.046 (2)	0.041 (2)	0.0013 (19)	0.0017 (19)	−0.0084 (17)
N2	0.055 (2)	0.0399 (18)	0.042 (2)	−0.0044 (16)	0.0069 (17)	0.0000 (16)
N3	0.051 (2)	0.0357 (18)	0.0412 (19)	0.0074 (15)	0.0003 (16)	−0.0021 (15)
N4	0.055 (2)	0.0408 (19)	0.046 (2)	0.0010 (17)	0.0041 (17)	−0.0075 (16)
N5	0.047 (2)	0.0363 (18)	0.055 (2)	−0.0043 (16)	0.0056 (18)	0.0003 (16)
N6	0.085 (4)	0.064 (3)	0.073 (3)	0.003 (3)	0.010 (3)	0.001 (3)
O1	0.107 (3)	0.094 (3)	0.065 (2)	−0.038 (2)	0.010 (2)	0.014 (2)
O2	0.063 (2)	0.0417 (16)	0.0450 (16)	0.0120 (15)	−0.0001 (14)	−0.0061 (13)
O3	0.056 (2)	0.0510 (17)	0.0520 (18)	0.0099 (15)	−0.0090 (15)	−0.0051 (15)
O4	0.067 (2)	0.057 (2)	0.053 (2)	0.0065 (17)	−0.0120 (17)	−0.0105 (18)
O5	0.145 (5)	0.098 (3)	0.162 (5)	0.057 (3)	−0.007 (4)	−0.042 (3)
O6	0.154 (4)	0.086 (3)	0.080 (3)	0.009 (3)	0.029 (3)	−0.020 (3)
O7	0.113 (4)	0.108 (4)	0.137 (4)	−0.013 (3)	−0.025 (3)	0.016 (3)
C1	0.089 (5)	0.108 (5)	0.097 (5)	0.002 (4)	−0.011 (4)	−0.047 (4)
C2	0.090 (6)	0.198 (11)	0.138 (9)	0.035 (7)	−0.051 (7)	−0.090 (7)
C3	0.163 (12)	0.230 (13)	0.091 (7)	0.116 (11)	−0.047 (7)	−0.060 (7)
C4	0.167 (8)	0.162 (8)	0.054 (4)	0.085 (7)	0.013 (5)	0.024 (4)
C5	0.104 (5)	0.107 (5)	0.051 (3)	0.029 (4)	0.012 (3)	0.014 (3)
C6	0.065 (4)	0.079 (3)	0.047 (3)	0.005 (3)	−0.002 (2)	−0.024 (3)
C7	0.086 (4)	0.051 (3)	0.038 (2)	−0.011 (3)	0.003 (3)	−0.011 (2)
C8	0.076 (3)	0.048 (3)	0.048 (3)	0.010 (2)	−0.006 (2)	−0.007 (2)
C9	0.045 (3)	0.035 (2)	0.044 (2)	−0.0057 (19)	0.003 (2)	0.0005 (19)
C10	0.097 (4)	0.056 (3)	0.044 (3)	−0.005 (3)	0.011 (3)	0.005 (2)
C11	0.140 (6)	0.052 (3)	0.061 (3)	−0.009 (4)	0.044 (4)	0.008 (3)
C12	0.091 (5)	0.060 (3)	0.103 (5)	−0.017 (3)	0.055 (4)	−0.005 (3)
C13	0.060 (4)	0.049 (3)	0.097 (4)	−0.007 (2)	0.028 (3)	−0.011 (3)
C14	0.046 (3)	0.035 (2)	0.064 (3)	−0.0046 (19)	0.011 (2)	−0.010 (2)
C20	0.054 (4)	0.064 (4)	0.168 (7)	−0.014 (3)	0.031 (4)	−0.014 (4)
C15	0.043 (3)	0.037 (2)	0.059 (3)	0.0030 (18)	−0.002 (2)	−0.0172 (19)
C16	0.053 (3)	0.053 (3)	0.092 (4)	0.011 (2)	−0.016 (3)	−0.027 (3)
C17	0.084 (4)	0.073 (4)	0.077 (4)	0.031 (3)	−0.035 (3)	−0.032 (3)
C18	0.119 (5)	0.071 (4)	0.052 (3)	0.033 (4)	−0.018 (3)	−0.005 (3)
C19	0.080 (4)	0.055 (3)	0.047 (3)	0.011 (3)	−0.004 (3)	0.003 (2)
C21	0.043 (3)	0.071 (4)	0.155 (7)	−0.005 (3)	−0.009 (4)	−0.033 (4)
C22	0.080 (4)	0.054 (3)	0.058 (3)	0.000 (3)	0.013 (3)	−0.011 (2)
C23	0.106 (5)	0.072 (4)	0.062 (3)	0.018 (3)	0.017 (3)	−0.024 (3)
C24	0.103 (5)	0.057 (3)	0.067 (3)	0.023 (3)	−0.013 (3)	−0.026 (3)
C25	0.069 (3)	0.041 (2)	0.059 (3)	0.007 (2)	−0.020 (3)	−0.013 (2)
C26	0.050 (3)	0.037 (2)	0.046 (2)	0.0065 (19)	−0.016 (2)	−0.0072 (19)
C27	0.040 (3)	0.034 (2)	0.055 (3)	−0.0003 (18)	−0.009 (2)	0.0014 (19)
C28	0.049 (3)	0.045 (3)	0.067 (3)	−0.008 (2)	−0.013 (2)	0.009 (2)
C29	0.059 (3)	0.060 (3)	0.095 (4)	−0.009 (3)	0.003 (3)	0.029 (3)
C30	0.060 (3)	0.072 (3)	0.080 (4)	0.000 (3)	0.027 (3)	0.020 (3)
C31	0.067 (3)	0.053 (3)	0.062 (3)	0.001 (2)	0.021 (3)	0.003 (2)
C32	0.091 (4)	0.035 (2)	0.095 (4)	−0.002 (3)	−0.033 (4)	−0.010 (3)
C33	0.080 (4)	0.038 (3)	0.101 (4)	−0.019 (3)	−0.021 (3)	0.011 (3)
O8	0.228 (6)	0.099 (3)	0.070 (3)	0.078 (3)	−0.047 (3)	−0.014 (2)

### Geometric parameters (Å, °)

**Table:**

Co1—O2	2.076 (3)	C10—H10A	0.9300
Co1—O4	2.106 (4)	C11—C12	1.349 (8)
Co1—N5	2.130 (3)	C11—H11A	0.9300
Co1—N2	2.133 (3)	C12—C13	1.386 (8)
Co1—N3	2.142 (3)	C12—H12A	0.9300
Co1—N4	2.173 (3)	C13—C14	1.396 (6)
N1—C7	1.332 (6)	C13—C20	1.434 (8)
N1—C8	1.453 (6)	C14—C15	1.432 (6)
N1—H1G	0.86 (7)	C20—C21	1.340 (9)
N2—C10	1.335 (5)	C20—H20A	0.9300
N2—C14	1.345 (5)	C15—C16	1.404 (6)
N3—C19	1.322 (5)	C16—C17	1.385 (8)
N3—C15	1.363 (5)	C16—C21	1.433 (8)
N4—C22	1.320 (5)	C17—C18	1.362 (8)
N4—C26	1.352 (5)	C17—H17A	0.9300
N5—C31	1.324 (5)	C18—C19	1.403 (7)
N5—C27	1.363 (5)	C18—H18A	0.9300
N6—O5	1.192 (6)	C19—H19A	0.9300
N6—O6	1.219 (5)	C21—H21A	0.9300
N6—O7	1.241 (6)	C22—C23	1.383 (6)
O1—C7	1.230 (5)	C22—H22A	0.9300
O2—C9	1.248 (5)	C23—C24	1.365 (7)
O3—C9	1.248 (5)	C23—H23A	0.9300
O4—H4F	0.85 (7)	C24—C25	1.395 (7)
O4—H4E	0.87 (6)	C24—H24A	0.9300
C1—C2	1.386 (11)	C25—C26	1.409 (6)
C1—C6	1.390 (8)	C25—C32	1.430 (7)
C1—H1A	0.9300	C26—C27	1.442 (6)
C2—C3	1.342 (14)	C27—C28	1.395 (6)
C2—H2A	0.9300	C28—C29	1.410 (7)
C3—C4	1.335 (13)	C28—C33	1.423 (7)
C3—H3A	0.9300	C29—C30	1.348 (7)
C4—C5	1.403 (9)	C29—H29A	0.9300
C4—H4A	0.9300	C30—C31	1.396 (6)
C5—C6	1.384 (7)	C30—H30A	0.9300
C5—H5A	0.9300	C31—H31A	0.9300
C6—C7	1.498 (7)	C32—C33	1.339 (7)
C8—C9	1.524 (5)	C32—H32A	0.9300
C8—H8A	0.9700	C33—H33A	0.9300
C8—H8B	0.9700	O8—H8F	0.8504
C10—C11	1.412 (7)	O8—H8E	0.8689
			
O2—Co1—O4	86.91 (13)	C12—C11—C10	119.8 (5)
O2—Co1—N5	92.25 (12)	C12—C11—H11A	120.1
O4—Co1—N5	91.65 (14)	C10—C11—H11A	120.1
O2—Co1—N2	98.52 (12)	C11—C12—C13	119.6 (5)
O4—Co1—N2	95.91 (15)	C11—C12—H12A	120.2
N5—Co1—N2	167.14 (13)	C13—C12—H12A	120.2
O2—Co1—N3	82.63 (12)	C12—C13—C14	117.9 (5)
O4—Co1—N3	166.78 (13)	C12—C13—C20	122.8 (6)
N5—Co1—N3	96.80 (13)	C14—C13—C20	119.4 (6)
N2—Co1—N3	77.76 (13)	N2—C14—C13	123.0 (5)
O2—Co1—N4	168.91 (12)	N2—C14—C15	117.9 (4)
O4—Co1—N4	96.67 (14)	C13—C14—C15	119.1 (5)
N5—Co1—N4	77.20 (13)	C21—C20—C13	121.0 (6)
N2—Co1—N4	91.57 (13)	C21—C20—H20A	119.5
N3—Co1—N4	95.12 (12)	C13—C20—H20A	119.5
C7—N1—C8	119.9 (4)	N3—C15—C16	122.2 (4)
C7—N1—H1G	126 (5)	N3—C15—C14	117.1 (4)
C8—N1—H1G	111 (5)	C16—C15—C14	120.7 (4)
C10—N2—C14	118.1 (4)	C17—C16—C15	118.1 (5)
C10—N2—Co1	128.2 (3)	C17—C16—C21	123.8 (6)
C14—N2—Co1	113.7 (3)	C15—C16—C21	118.1 (5)
C19—N3—C15	117.9 (4)	C18—C17—C16	119.8 (5)
C19—N3—Co1	128.5 (3)	C18—C17—H17A	120.1
C15—N3—Co1	112.9 (3)	C16—C17—H17A	120.1
C22—N4—C26	117.4 (4)	C17—C18—C19	119.0 (5)
C22—N4—Co1	129.1 (3)	C17—C18—H18A	120.5
C26—N4—Co1	113.5 (3)	C19—C18—H18A	120.5
C31—N5—C27	117.8 (4)	N3—C19—C18	122.9 (5)
C31—N5—Co1	127.3 (3)	N3—C19—H19A	118.5
C27—N5—Co1	114.8 (3)	C18—C19—H19A	118.5
O5—N6—O6	121.9 (6)	C20—C21—C16	121.6 (6)
O5—N6—O7	120.0 (6)	C20—C21—H21A	119.2
O6—N6—O7	118.0 (6)	C16—C21—H21A	119.2
C9—O2—Co1	134.3 (3)	N4—C22—C23	123.6 (5)
Co1—O4—H4F	96 (5)	N4—C22—H22A	118.2
Co1—O4—H4E	126 (4)	C23—C22—H22A	118.2
H4F—O4—H4E	99 (6)	C24—C23—C22	119.0 (5)
C2—C1—C6	121.0 (8)	C24—C23—H23A	120.5
C2—C1—H1A	119.5	C22—C23—H23A	120.5
C6—C1—H1A	119.5	C23—C24—C25	120.2 (5)
C3—C2—C1	119.6 (11)	C23—C24—H24A	119.9
C3—C2—H2A	120.2	C25—C24—H24A	119.9
C1—C2—H2A	120.2	C24—C25—C26	116.3 (5)
C4—C3—C2	121.3 (11)	C24—C25—C32	124.7 (5)
C4—C3—H3A	119.3	C26—C25—C32	119.0 (5)
C2—C3—H3A	119.3	N4—C26—C25	123.5 (4)
C3—C4—C5	120.9 (10)	N4—C26—C27	117.5 (4)
C3—C4—H4A	119.5	C25—C26—C27	118.9 (4)
C5—C4—H4A	119.5	N5—C27—C28	123.3 (4)
C6—C5—C4	119.2 (7)	N5—C27—C26	116.9 (4)
C6—C5—H5A	120.4	C28—C27—C26	119.8 (4)
C4—C5—H5A	120.4	C27—C28—C29	116.5 (4)
C5—C6—C1	118.0 (6)	C27—C28—C33	119.8 (5)
C5—C6—C7	123.6 (5)	C29—C28—C33	123.7 (5)
C1—C6—C7	118.4 (6)	C30—C29—C28	120.3 (5)
O1—C7—N1	122.1 (5)	C30—C29—H29A	119.9
O1—C7—C6	120.5 (5)	C28—C29—H29A	119.9
N1—C7—C6	117.4 (4)	C29—C30—C31	119.5 (5)
N1—C8—C9	114.0 (3)	C29—C30—H30A	120.2
N1—C8—H8A	108.8	C31—C30—H30A	120.2
C9—C8—H8A	108.8	N5—C31—C30	122.6 (5)
N1—C8—H8B	108.8	N5—C31—H31A	118.7
C9—C8—H8B	108.8	C30—C31—H31A	118.7
H8A—C8—H8B	107.7	C33—C32—C25	121.7 (5)
O3—C9—O2	126.5 (4)	C33—C32—H32A	119.1
O3—C9—C8	115.8 (4)	C25—C32—H32A	119.1
O2—C9—C8	117.7 (4)	C32—C33—C28	120.7 (5)
N2—C10—C11	121.5 (5)	C32—C33—H33A	119.7
N2—C10—H10A	119.3	C28—C33—H33A	119.7
C11—C10—H10A	119.3	H8F—O8—H8E	119.3
			
O2—Co1—N2—C10	105.1 (4)	C11—C12—C13—C20	177.3 (5)
O4—Co1—N2—C10	17.4 (4)	C10—N2—C14—C13	−2.4 (6)
N5—Co1—N2—C10	−108.3 (7)	Co1—N2—C14—C13	178.8 (3)
N3—Co1—N2—C10	−174.4 (4)	C10—N2—C14—C15	178.0 (4)
N4—Co1—N2—C10	−79.5 (4)	Co1—N2—C14—C15	−0.8 (5)
O2—Co1—N2—C14	−76.3 (3)	C12—C13—C14—N2	4.1 (7)
O4—Co1—N2—C14	−164.0 (3)	C20—C13—C14—N2	−175.7 (4)
N5—Co1—N2—C14	70.3 (7)	C12—C13—C14—C15	−176.3 (4)
N3—Co1—N2—C14	4.2 (3)	C20—C13—C14—C15	3.9 (6)
N4—Co1—N2—C14	99.1 (3)	C12—C13—C20—C21	179.6 (5)
O2—Co1—N3—C19	−77.2 (3)	C14—C13—C20—C21	−0.6 (8)
O4—Co1—N3—C19	−115.2 (7)	C19—N3—C15—C16	−0.5 (6)
N5—Co1—N3—C19	14.2 (4)	Co1—N3—C15—C16	−172.1 (3)
N2—Co1—N3—C19	−177.6 (4)	C19—N3—C15—C14	−179.3 (4)
N4—Co1—N3—C19	91.9 (4)	Co1—N3—C15—C14	9.1 (4)
O2—Co1—N3—C15	93.3 (3)	N2—C14—C15—N3	−5.8 (5)
O4—Co1—N3—C15	55.3 (7)	C13—C14—C15—N3	174.7 (4)
N5—Co1—N3—C15	−175.3 (3)	N2—C14—C15—C16	175.4 (4)
N2—Co1—N3—C15	−7.1 (3)	C13—C14—C15—C16	−4.2 (6)
N4—Co1—N3—C15	−97.6 (3)	N3—C15—C16—C17	0.6 (6)
O2—Co1—N4—C22	160.5 (6)	C14—C15—C16—C17	179.4 (4)
O4—Co1—N4—C22	−91.2 (4)	N3—C15—C16—C21	−177.6 (4)
N5—Co1—N4—C22	178.6 (4)	C14—C15—C16—C21	1.1 (6)
N2—Co1—N4—C22	4.9 (4)	C15—C16—C17—C18	−1.1 (7)
N3—Co1—N4—C22	82.8 (4)	C21—C16—C17—C18	177.1 (5)
O2—Co1—N4—C26	−20.5 (8)	C16—C17—C18—C19	1.4 (8)
O4—Co1—N4—C26	87.8 (3)	C15—N3—C19—C18	0.8 (6)
N5—Co1—N4—C26	−2.3 (3)	Co1—N3—C19—C18	170.9 (3)
N2—Co1—N4—C26	−176.0 (3)	C17—C18—C19—N3	−1.3 (8)
N3—Co1—N4—C26	−98.2 (3)	C13—C20—C21—C16	−2.5 (9)
O2—Co1—N5—C31	−4.4 (4)	C17—C16—C21—C20	−175.9 (5)
O4—Co1—N5—C31	82.6 (4)	C15—C16—C21—C20	2.3 (8)
N2—Co1—N5—C31	−151.3 (6)	C26—N4—C22—C23	−0.2 (7)
N3—Co1—N5—C31	−87.2 (4)	Co1—N4—C22—C23	178.8 (4)
N4—Co1—N5—C31	179.1 (4)	N4—C22—C23—C24	−0.3 (9)
O2—Co1—N5—C27	179.4 (3)	C22—C23—C24—C25	0.7 (8)
O4—Co1—N5—C27	−93.6 (3)	C23—C24—C25—C26	−0.5 (7)
N2—Co1—N5—C27	32.5 (8)	C23—C24—C25—C32	−179.1 (5)
N3—Co1—N5—C27	96.6 (3)	C22—N4—C26—C25	0.3 (6)
N4—Co1—N5—C27	2.8 (3)	Co1—N4—C26—C25	−178.8 (3)
O4—Co1—O2—C9	17.3 (4)	C22—N4—C26—C27	−179.3 (4)
N5—Co1—O2—C9	108.9 (4)	Co1—N4—C26—C27	1.6 (4)
N2—Co1—O2—C9	−78.2 (4)	C24—C25—C26—N4	0.0 (6)
N3—Co1—O2—C9	−154.6 (4)	C32—C25—C26—N4	178.6 (4)
N4—Co1—O2—C9	126.5 (7)	C24—C25—C26—C27	179.6 (4)
C6—C1—C2—C3	−0.6 (13)	C32—C25—C26—C27	−1.8 (6)
C1—C2—C3—C4	0.6 (16)	C31—N5—C27—C28	−1.2 (6)
C2—C3—C4—C5	−0.2 (15)	Co1—N5—C27—C28	175.4 (3)
C3—C4—C5—C6	−0.1 (11)	C31—N5—C27—C26	−179.6 (4)
C4—C5—C6—C1	0.0 (8)	Co1—N5—C27—C26	−3.0 (5)
C4—C5—C6—C7	−177.6 (5)	N4—C26—C27—N5	0.9 (5)
C2—C1—C6—C5	0.4 (8)	C25—C26—C27—N5	−178.7 (4)
C2—C1—C6—C7	178.1 (6)	N4—C26—C27—C28	−177.5 (4)
C8—N1—C7—O1	−11.4 (6)	C25—C26—C27—C28	2.8 (6)
C8—N1—C7—C6	170.5 (4)	N5—C27—C28—C29	0.8 (6)
C5—C6—C7—O1	167.1 (5)	C26—C27—C28—C29	179.1 (4)
C1—C6—C7—O1	−10.5 (7)	N5—C27—C28—C33	179.4 (4)
C5—C6—C7—N1	−14.8 (7)	C26—C27—C28—C33	−2.3 (6)
C1—C6—C7—N1	167.6 (4)	C27—C28—C29—C30	−0.4 (7)
C7—N1—C8—C9	−77.2 (5)	C33—C28—C29—C30	−179.0 (5)
Co1—O2—C9—O3	−4.7 (7)	C28—C29—C30—C31	0.5 (8)
Co1—O2—C9—C8	175.5 (3)	C27—N5—C31—C30	1.3 (7)
N1—C8—C9—O3	177.4 (4)	Co1—N5—C31—C30	−174.9 (4)
N1—C8—C9—O2	−2.8 (6)	C29—C30—C31—N5	−1.0 (8)
C14—N2—C10—C11	−0.8 (6)	C24—C25—C32—C33	178.6 (5)
Co1—N2—C10—C11	177.8 (3)	C26—C25—C32—C33	0.1 (7)
N2—C10—C11—C12	2.3 (8)	C25—C32—C33—C28	0.5 (8)
C10—C11—C12—C13	−0.6 (8)	C27—C28—C33—C32	0.6 (7)
C11—C12—C13—C14	−2.4 (8)	C29—C28—C33—C32	179.1 (5)

### Hydrogen-bond geometry (Å, °)

**Table:**

*D*—H···*A*	*D*—H	H···*A*	*D*···*A*	*D*—H···*A*
O8—H8E···O5	0.87	2.46	3.084 (7)	129
O8—H8E···O7	0.87	2.24	2.982 (7)	143
O4—H4F···O2	0.85 (7)	2.48 (7)	2.876 (5)	110 (5)
O4—H4F···O3	0.85 (7)	1.87 (7)	2.696 (5)	162 (7)
